# Probabilistic approach for assessing cancer risk due to benzo[*a*]pyrene in barbecued meat: Informing advice for population groups

**DOI:** 10.1371/journal.pone.0207032

**Published:** 2018-11-08

**Authors:** Lea Sletting Jakobsen, Stylianos Georgiadis, Bo Friis Nielsen, Bas G. H. Bokkers, Elena Boriani, Lene Duedahl-Olesen, Tine Hald, Maarten J. Nauta, Anders Stockmarr, Sara M. Pires

**Affiliations:** 1 National Food Institute, Technical University of Denmark, Kgs. Lyngby, Denmark; 2 Department of Applied Mathematics and Computer Science, Technical University of Denmark, Kgs. Lyngby, Denmark; 3 National Institute for Public Health and the Environment (RIVM), Bilthoven, Netherlands; Epidstat Institute, UNITED STATES

## Abstract

**Background:**

Consumption of meat prepared by barbecuing is associated with risk of cancer due to formation of carcinogenic compounds including benzo[*a*]pyrene (BaP). Assessment of a population’s risk of disease and people’s individual probability of disease given specific consumer attributes may direct food safety strategies to where impact on public health is largest. The aim of this study was to propose a model that estimates the risk of cancer caused by exposure to BaP from barbecued meat in Denmark, and to estimate the probability of developing cancer in subgroups of the population given different barbecuing frequencies.

**Methods:**

We developed probabilistic models applying two dimensional Monte Carlo simulation to take into account the variation in exposure given age and sex and in the individuals’ sensitivity to develop cancer after exposure to BaP, and the uncertainty in the dose response model. We used the Danish dietary consumption survey, monitoring data of chemical concentrations, data on consumer behavior of frequency of barbecuing, and animal dose response data.

**Findings:**

We estimated an average extra lifetime risk of cancer due to BaP from barbecued meat of 6.8 × 10^−5^ (95% uncertainty interval 2.6 × 10^−7^ − 7.0 × 10^−4^) in the Danish population. This corresponds to approximately one to 4,074 extra cancer cases over a lifetime, reflecting wide uncertainty. The impact per barbecuing event on the risk of cancer for men and women of low body weight was higher compared to higher bodyweight. However, the difference due to sex and bodyweight between subgroups are dwarfed by the uncertainty.

**Interpretation:**

This study proposes a model that can be applied to other substances and routes of exposure, and allows for deriving the change in risk following a specific change in behaviour. The presented methodology can serve as a valuable tool for risk management, allowing for the formulation of behaviour advice targeted to specific sub-groups in the population.

## Introduction

Based on an assessment of the available scientific literature, the International Agency for Research on Cancer concluded in 2015 that consumption of processed meat increases the risk of cancer in humans [1]. Processing of meat corresponds to production or cooking practices in which meat is transformed to enhance organoleptic properties, digestibility and preservation including smoking, curing and various heat treatments. The compounds that are considered responsible for the carcinogenicity of processed meat are formed during these processes [2], and include polycyclic aromatic hydrocarbons (PAHs) that constitute a large group of compounds that are formed during incomplete combustion of organic matter. If meat is prepared over open flame (e.g. barbecuing), fat or meat-juice drips onto the hot coals, wood, etc., and PAHs, formed in the smoke, adhere to the surface of the meat [3, 4]. Sixteen PAHs have been found to be genotoxic/mutagenic and/or carcinogenic in toxicological studies [5–7], benzo[*a*]pyrene (BaP) being the most studied PAH and classified as carcinogenic to humans (group 1) by a genotoxic mode of action [8].

To limit the population’s exposure to BaP and other carcinogenic PAHs from barbecued meat, the European Commission has implemented official mitigation strategies that include a legally enforced maximum limit of 5 μg/kg in commercial prepared heat treated meat [9], monitoring of concentration of PAH in meat barbecued in restaurants or other commercial settings, and guidance on how to adjust processing to decrease contamination. In Denmark, the National Food Authority further advises the population to limit consumption of barbecued meat and if barbecuing, to avoid charred meat [10]. Current mitigation strategies are commonly based on deterministic approaches to risk assessment. These define variables as point estimates, usually conservative estimates, aimed at ensuring protection of the population. However, the output of such risk assessment does not provide information on which subgroups of the population could be higher risk, or of the variability and uncertainty of these estimates [11]

To allow risk managers to act with precision, the variability (the inherent difference between e.g. individuals in a population, concentrations in samples or any other property of the system being studied) must be described, and the impact of variability on the population’s and the individual’s risk of disease quantified. At the same time, quantification of the uncertainty (the lack of knowledge of a true value or relationship between values) in the risk estimates allows risk managers to judge how probable the size of the risk is for individuals in the population.

Additionally, in the case of food processing practices, consumers are ultimately their own risk managers and access to information on the probability of a harmful effect given their food consumption patterns will allow them to make more informed choices on food consumption behavior, e.g. frequency of consumption of barbecued meat [12].

Stochasticity may be the option to provide this information by describing the parameters in the risk assessment algorithm that are variable and/or uncertain by probability distributions [11]. Probabilistic risk assessment in food safety is an established and applied research area in quantitative microbial risk assessment [13] and in exposure assessments to chemicals [14, 15]. Probabilistic chemical risk assessments are still emerging, with methodologies developed such as the Integrated Probabilistic Risk Assessment(IPRA) [16–18] and tools such as the Aproximate Probabilistic-tool (APROBA) [19–21]. These take into account the uncertainty and variability in order to quantify the degree of conservatism in risk assessments for regulatory purposes.

The aim of this study was to develop a probabilistic model to estimate the extra lifetime risk (in terms of increased probability) of exposure to chemical contaminants in food, using BaP exposure from consumption of barbecued meat and fish as a case study, applying the available data representative of the Danish population. The specific objectives were to estimate the extra lifetime risk of cancer caused by the exposure to BaP from barbecued meat; 1) in the total adult Danish population and 2) in subgroups of the population and as a function of the frequency of events of consuming barbecued meat.

We took into account the variation in consumer behaviour and the variation in individual’s sensitivity to develop cancer after exposure to BaP, as well as the uncertainty in the conversion between the human and animal species and in the dose response relationship. We applied second order Monte Carlo simulation in order to keep separated the uncertainty domain from the variability domain, as they are inherently different properties, which must be taken into account in the interpretation of the results [11, 22].

## Materials and methods

**Fig 1:**
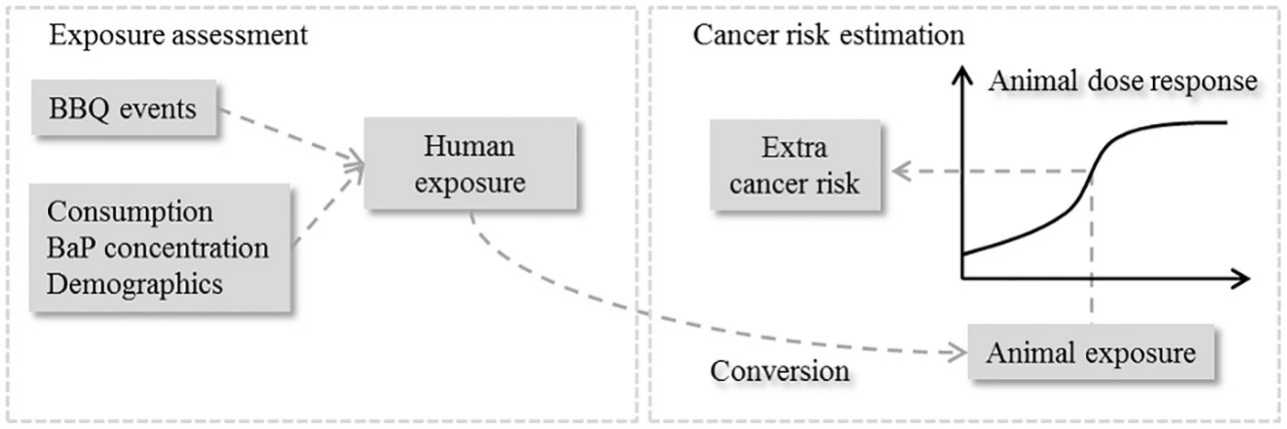
Conceptual overview of the risk model. The human exposure to benzo[*a*]pyrene (BaP) from barbecued meat consumed per event of barbecuing (BBQ event) was converted to an equivalent animal exposure in order to be combined with a dose-response relationship obtained from an animal study to estimate the extra lifetime risk of developing cancer.

To address both objectives, we applied an event-based simulation scheme for the exposure assessment and a model extrapolation approach for estimating the risk of developing cancer. Fig 1 presents an overview of the model-structure. The data used and the modelling approaches applied for the exposure assessment and cancer risk estimation are described in the following. All algorithms and simulations were developed and performed in R version 3.3.1 [23].

### Exposure assessment

In the following, the data and modelling approaches applied for the exposure assessment is described.

#### Meat consumption

**Fig 2:**
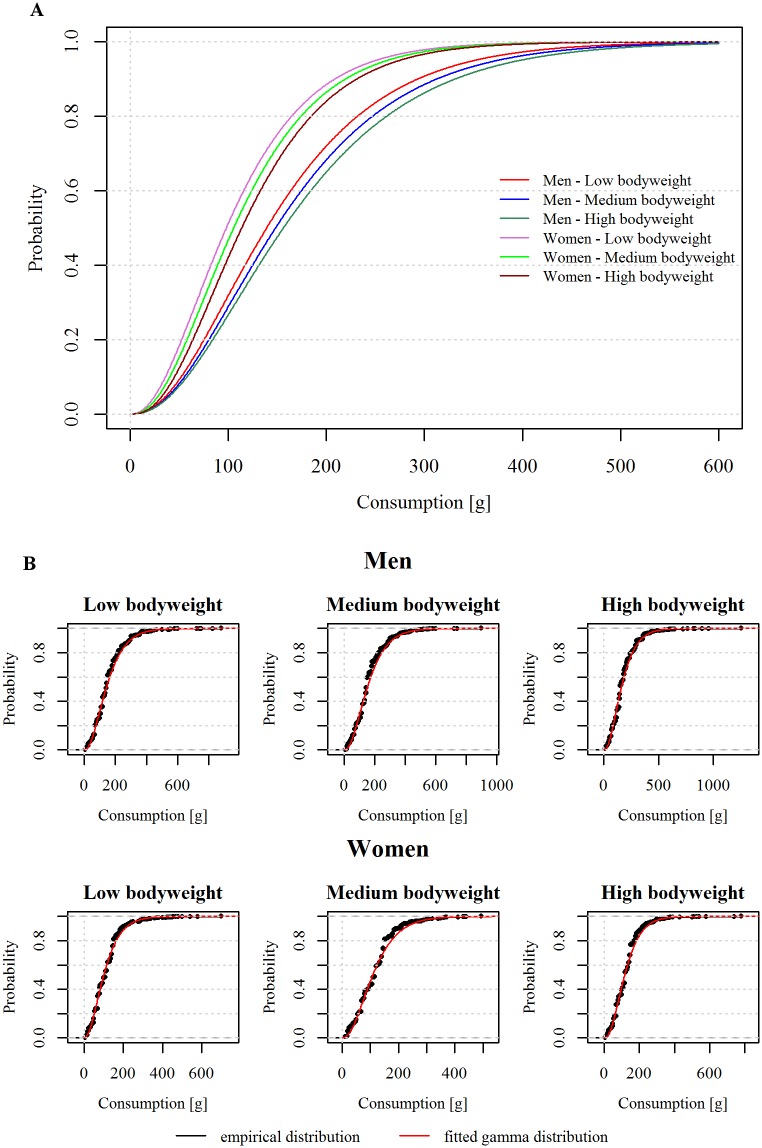
Cumulative distribution functions of meat consumption in g/meal for each sex and weight class. A: The theoretical cumulative distribution functions of the fitted gamma distributions. B: The empirical and theoretical cumulative distribution functions of meat consumption for each sex and weight class. The red line in each graph represents the gamma distribution fitted to the meat consumptions. The black lines depict the empirical distributions and the black marks represent the observed consumed amounts of meat.

We accounted for exposure to BaP through both meat and fish consumption. For simplicity, we refer to those as barbecued “meat”. Meat consumption data were obtained from the Danish National Survey on Diet and Physical Activity (DANSDA) from 2011-2013 [24], consisting of 7 day food-records from 3,804 individuals, along with sex, age (4-75 years old) and bodyweight for each individual. This survey is considered representative of the Danish population. We considered the food consumption of the adult population, i.e. from 16 years old and up to 75; a total of 1,461 men and 1,572 women. Individual meat consumption in DANSDA are given for 6 meals per day, but we only considered meat consumptions from dinner-eating occasions, which is typically a warm meal, as a proxy for lunch or dinner. We assumed that consumption of barbecued meat occurs in at most one meal per day. Only food consumption of individuals with reported bodyweight and only dinners with non-zero total consumption were considered. Each individual in the survey was assigned to a weight-class based on the 33^rd^ and 67^th^ quantiles of the observed body-weights in the consumption survey (Table 1). The number of weight classes (three) was chosen based on relevant group sizes from the study data, i.e. further subdivision would limit the number of participants in each class. For each weight class, the meat consumption in g/meal was described by a distinct gamma distribution (S1 Table). The empirical and fitted gamma cumulative density functions of each meat type are shown in Fig 2. To allocate a simulated individual to a weight-class, the relation between age, sex and bodyweight was evaluated based on the data in DANSDA. Different options for the relation were evaluated and the following was selected:

$$\begin{array}{c} \text{weight}=\beta_{1}\times \text{sex}+\beta_{2}\times \text{age}+\beta_{3}\times \ln\left(\text{age}\right)+\epsilon , \end{array}$$ (1)

where *β*_1_ = −14.476, *β*_2_ = −0.829 and *β*_3_ = 36.406 (adjusted R-squared is 0.971 and residual standard error is 13.265). The error term, *ϵ*, is assumed to follow a normal distribution. The weight of an individual was simulated through Eq (1), with the standard deviation of the error term in the regression formula estimated by the residual standard error.

**Table 1:** Classes of bodyweight (in kg).

Weight class	Men bodyweight in kg (median of weight class)	Women bodyweight in kg (median of weight class)
Low	≤ 76(71)	≤ 62(58)
Medium	77 − 87(82)	63 − 72(67)
High	≥ 88(96)	≥ 73(80)

Weight classes defined by the 33^rd^ and 67^th^ quantiles of the bodyweight of individuals aged 16-75 in the Danish National Survey on Diet and Physical Activity.

#### Combination of meat types consumed

For each event of consuming barbecued meat, we assumed that one individual would eat a maximum of two different meat types (S2 Table). In order to derive the frequency of type of meat consumed if only one meat is consumed (S3 Table) or the possible meat combinations as well as the partial consumption when two meat types are consumed (S4 Table), the consumption data were coupled with a survey conducted by Coop Denmark A/S in 2013 with 1,009 Danish respondents aged between 15 and 74 years [25].

#### Frequency of consumption of barbecued meat

We assumed that all individuals in the Danish population who eat meat, eat barbecued meat at least once a year. Thus, the fraction of the population never consuming barbecued meat is the same as that of never eating meat, which was estimated to be 4% based on a consumer survey by Coop Denmark A/S [26]. We obtained information on the frequency of consuming barbecued meat from a consumer survey on barbecuing behaviour of 100 Danish households from a suburb to Copenhagen, Denmark [27]. Families consisted of 2-6 people each, either adults only or adults with children, an overall age range of 1-89 years, and equal distribution of men (50.3%) and women (49.7%). No information on the bodyweight of the participants was provided. Out of the 100 households, 76 (equal to 189 individuals) provided qualitative information on how often the household was barbecuing, which we translated into an annual number of consumption events. The scenarios for barbecuing frequency that we considered for the Danish population were: 1) less than once per month, i.e. 1-11 times per year; 2) at least once per month but less than once per week, i.e. 12-51 times per year; 3) at least once per week, i.e. 52-365 times per year. The median value for each scenario was selected to represent the scenario. The fraction of the Danish population belonging to each scenario was estimated based on the data from the 76 families (Table 2).

**Table 2:** Frequency of events of consuming barbequed meat per year for fractions of the Danish population.

Frequency of consuming barbecued meat (events per year)	0	9	25	63
Fraction of population	0.040	0.265	0.455	0.240

Derived from consumer-surveys in Denmark [26, 27].

#### Concentration of BaP in meat types

**Fig 3:**
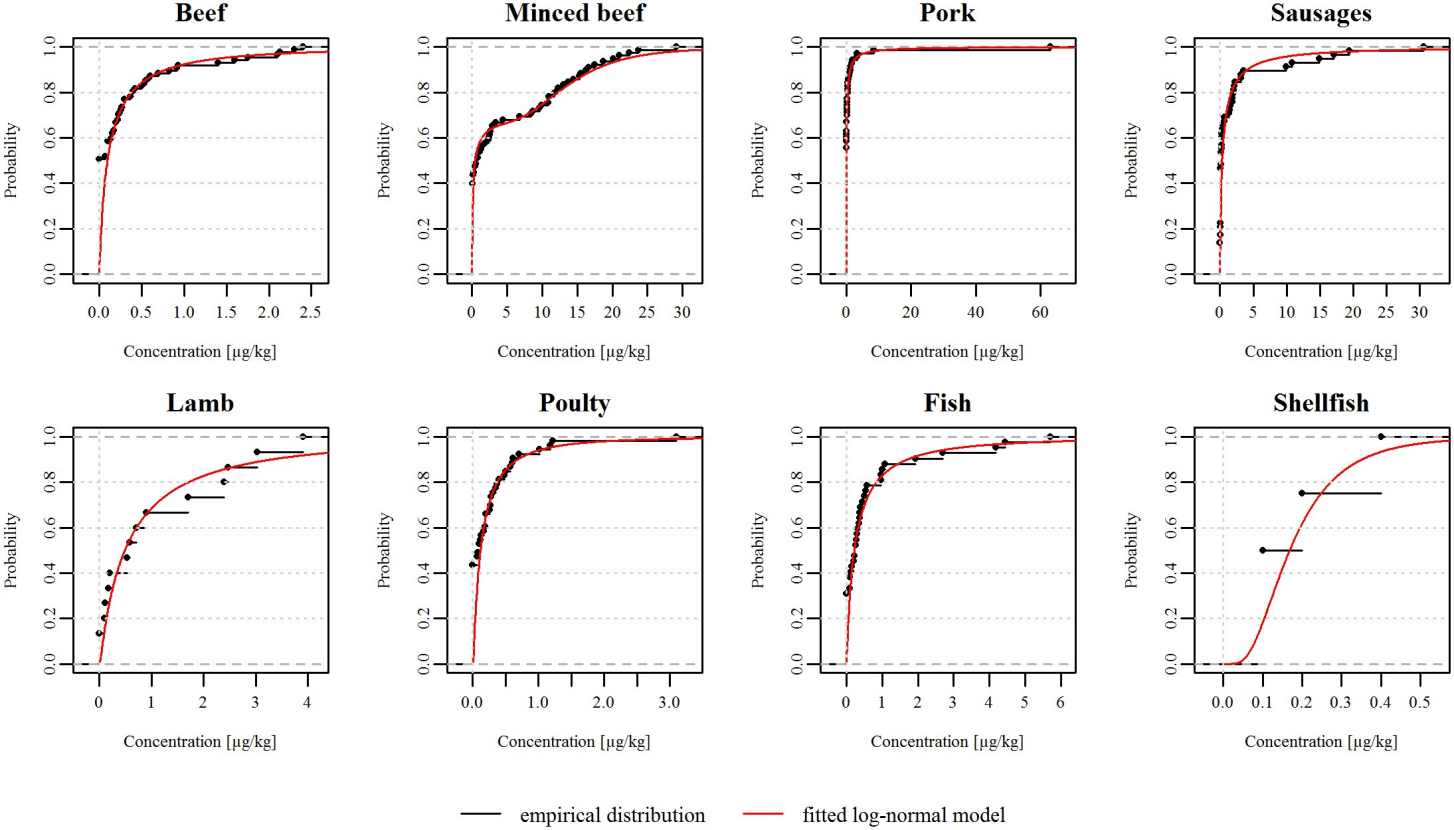
Empirical and theoretical cumulative distribution functions for concentration of benzo[*a*]pyrene in each meat type. The red lines in each graph represent the log-normal distributions fitted to the concentrations. The black lines depict the empirical distributions and the black marks represent the observed concentrations of benzo[*a*]pyrene (in *μg*/*kg*) of each sample of each meat type.

The concentration of BaP in meat after barbecuing (in *μ*g/kg) was obtained for 407 samples of meat (242 from Denmark obtained from [28] and from unpublished monitoring data of commercially barbecued meat from 2012-2015; 136 from UK [29, 30]; and 29 from Sweden [31]). Eight meat and fish types were considered: beef (including veal), minced beef (burger patty), pork, pork sausages, lamb, poultry (mainly chicken), fish (mainly salmon) and shellfish. The concentration data from each country were combined for each food type, assuming that the data then reflects the variation in concentration of BaP under different conditions. Values for many samples were below the limit of detection (LOD) of the analytical method applied. After barbecuing all foods will be contaminated with BaP [32]. Therefore, apparent zeros were not regarded as “true zeros”, but rather as an expression of a low level of contamination between 0 *μ*g/kg and the LOD. Note that LoD is not unique, but it varies across samples. The BaP concentrations for each food type were consequently described by a log-normal distribution as depicted in Fig 3 (S5 Table). To estimate the parameters of the log-normal distributions, we formulated the likelihood function taking the censoring at the various LODs into account [33]. However, for minced beef, we applied a 2-component lognormal mixture model on the censored data, since a single component lognormal described the data inadequately [34]. The maximum a posteriori estimation was used to assess the parameters of the 2-component lognormal mixture model, choosing appropriate priors.

#### Demographics

To estimate the population exposure, we generated the sex and age distribution of the Danish population aged 16-75 years from the official statistics for the fourth quarter of 2017 (S1 Fig).

#### Modelling exposure

To derive the population or subgroup risk, we estimated the yearly BaP exposure *y*_*i*_ (in *μg*/*kg* bodyweight) through simulation of *B*_*i*_ barbecue events per year and per individual *i* by:

$$\begin{array}{c} y_{i}=\frac{1}{w_{i}}\sum_{b=1}^{B_{i}}\sum_{k=1}^{K_{ib}}x_{ibk}c_{ibk}, \end{array}$$ (2)

where *K*_*ib*_ is the total number (*K*_*ib*_ = 1, 2) of meat types consumed during a barbecue event *b* (*b* = 1, …, *B*_*i*_) by individual *i*; *x*_*ibk*_ is the amount of meat *k* (*k* = 1, …, *K*_*ib*_) consumed by individual *i* at event *b*; *c*_*ibk*_ is the concentration of BaP in meat *k* consumed by individual *i* at event *b*; and *w*_*i*_ is the bodyweight of individual *i*.

To simulate the Danish population, we generated the sex and age of individuals from the demographics data (S1 Fig). The bodyweight of the individuals was simulated through the regression model (Eq 1) and the individuals were assigned to a weight class (Table 1). For each individual, a number of barbecue events *B*_*i*_ among the possible scenarios was simulated (Table 2). For each barbecue event the consumed meat types and their partial consumption (S2, S3 and S4 Tables) were simulated independently of the individual’s weight class, while the total meat consumption was generated from the gamma distribution (S1 Table) defined for the weight class of the individual. The BaP concentration was randomly sampled from the log-normal distribution of the consumed meat types in (S5 Table). We simulated the exposure of 10,000 individuals to make up the population exposure distribution.

To estimate the exposure in the subgroups model as a function of the frequency of consuming barbecued meat, we also applied Eq (2), but now for each value of *B*_*i*_ from 1 to 100 (i.e. one event of consuming barbecued meat per year to 100 events per year). These simulations were performed for a “typical” individual of each weight class (low, medium, high) of each sex (i.e. 6 different subgroups), where the median bodyweight of each weight class (Table 1) represents the bodyweight *w*_*i*_ (Eq 2) of an individual of this weight class. Similar to the population model, the total meat consumption of the individual was generated from the appropriate gamma distribution, along with the consumed meat types and their partial consumption, and the BaP concentration from the appropriate log-normal distribution. The yearly exposure was simulated for 5,000 individuals of each weight class.

### Estimation of the extra lifetime risk of cancer

We applied a model extrapolation approach based on the integrated probabilistic risk assessment methodology for carcinogens [18, 35], to estimate the extra lifetime risk (in terms of increased probability) of cancer due to exposure to BaP from barbecued meat. Model extrapolation refers to the extrapolation of a dose response relationship beyond the observable range of doses used in the given dose response study. In the following, the data and modelling approaches applied for the cancer risk estimation is described.

#### Intra- and inter-species conversion

In order to combine the human exposure (expressed as rate in *μg*/*kg* bodyweight) with an animal dose response relationship, the human exposure was converted to an equivalent animal exposure rate. In the conversion, intra- and interspecies difference was taken into account by multiplying the human exposure, y_*i*_, with conversion factors (CF) (also referred to elsewhere as extrapolation-, assessment- or uncertainty factors) describing these differences:

$$\begin{array}{c} {\text{exp}}_{\text{animal},i}=y_{i}\times {\text{CF}}_{\text{intra},i}\times {\text{CF}}_{\text{inter},\text{allometric}}\times {\text{CF}}_{\text{inter},\text{TKTD}}, \end{array}$$ (3)

where exp_animal,*i*_ is the animal exposure corresponding to individual *i*, CF_intra,*i*_ is a conversion factor taking into account the inter-individual differences in the human population, CF_inter,allometric_ and CF_inter,TKTD_ are conversion factors taking into account the interspecies difference between humans and the animal species, mice in this study (see section below for details).

#### Conversion factors

Conversion factors were used to express the animal exposure, exp_animal,*i*_, converted from the human exposure, y_*i*_. The conversion factors were described by probability distributions (Table 3). The intraspecies conversion factor (CF_intra_) accounts for the variation in sensitivity to the chemical between individuals in the human population. Since the size of the intraspecies variation is generally unknown, the geometric standard deviation, GSD_CF_intra__ is considered uncertain, which is reflected by a distribution around the GSD [18]. The interspecies difference is performed in two steps; 1) allometric scaling to correct the exposure that is expressed as a rate (i.e. dose per kg bodyweight) for differences in bodysize between humans and animals (CF_inter,allometric_) by:

$$\begin{array}{c} {\text{CF}}_{\text{inter},\text{allometric}}={\left(\frac{{\text{bw}}_{\text{human}}}{{\text{bw}}_{\text{animal}}}\right)}^{1-\text{AP}}, \end{array}$$ (4)

where bw_human_ is the median of the bodyweights reported in DANSDA for either the overall population or the defined subgroups, bw_animal_ is the weight of a mouse and assumed to be 30 grams, and AP is the allometric power [18], which is considered to be uncertain and expressed by a probability distribution. The second step accounts for the remaining interspecies difference relating to differences in toxicokinetics and -dynamics (CF_inter,TKTD_) between humans and animals, which is also assumed to be uncertain [18]. Due to lack of substance specific estimates, default distributions for CF_intra_, CF_inter,allometric_ and CF_inter,TKTD_ were applied [18] (Table 3).

**Table 3:** Model parameters and distributional assumptions.

	Description	Unit	Distributional assumption	Distribution parameters	
				Population	Subgroups
CF_intra_ ^(1^	Variation in sensitivity to the chemical between individuals		Lognormal(GM,GSD)^(3^	GM_CF_intra__ = 1; GSD_CF_intra__ = 3.6	
GSD_CF_intra__ ^(1^	Uncertainty in the GSD of CF_intra_		Chi-squared(df)	df = 21	
AP^(1^	Uncertainty in AP		Normal(*μ*, *σ*^2^)	*μ* = 0.7; *σ* = 0.033	
CF_inter,TKTD_ ^(1^	Uncertainty in TKTD difference between human and animal		Lognormal(GM,GSD)^(2^	GM_CF_inter,TKTD__ = 1; GSD_CF_inter,TKTD__ = 2	
bw_human_	Human bodyweight	kg	Point estimate	76	subgroup medians^(3^
bw_animal_	Animal bodyweight	kg	Point estimate	0.03^(4^	
*b*	Potency parameter of the two-stage model		Empirical	(8629.8, 29089.6, 13937910)^(5^	(9025.3, 30344.3, 14751525.2)^(5^
*c*	Shape parameter of the two-stage model		Empirical	(0.18, 8.925, 3125271)^(5^	(0.26975, 9.785, 2691014)^(5^

^(1^ Default distributions used, when no chemical specific information is available [18].

^(2^ GM = geometric mean, GSD = geometric standard deviation.

^(3^ bw_human_ for each subgroup is given in Table 1.

^(4^ 0.03 kg is the assumed weight of a mouse.

^(5^ distribution values for the empirical distribution in brackets: (2.5^th^ percentile, median, 97.5^th^ percentile)

#### Dose-response relationship

To derive the relationship between exposure to BaP and the risk of developing cancer, we used data on tumor formation in mice orally exposed to either of two coal tar mixtures [36]. The BaP content measured in the coal tar mixtures [37] was used as the dose. The number of all tumor bearing animals was used as response variable (S6 Table). Hence, we assumed that the composition of the PAH mixture present in barbecued meat is similar to the composition of PAH in the coal tar mixture, and subsequently that BaP is a surrogate for the total potency of all PAHs present in the coal tar mixtures. We performed dose-response modelling on the data using PROAST version 65.5 [38] developed in R [23]. The usual suit of models were fitted to the data and of the models accepted [39], we chose the most sensitive to estimate the extra lifetime risk, ER_BaP_, associated with the lifetime exposure to BaP, i.e. the model yielding the lowest BMDL_10_ (the benchmark dose lower limit, i.e. the 5^th^ percentile of the distribution of doses at which 10% of the study animals get a tumor), which was the two-stage model:

$$\begin{array}{c} {\text{ER}}_{\text{BaP}}=1-(e^{\left(\frac{{\text{exp}}_{\text{animal}}}{b}\right)-c{\left(\frac{{\text{exp}}_{\text{animal}}}{b}\right)}^{2}}), \end{array}$$ (5)

where ER_BaP_ is the extra lifetime risk of cancer due to BaP, exp_animal_ is the animal exposure, *b* is the potency parameter and *c* is the shape parameter (Table 3). The model parameters *b* and *c* were generated by bootstrapping within PROAST [38].

#### Modelling risk

As the human exposure distribution represents variability between individuals, CF_inter,allometric_, CF_inter,TKTD_ and two-stage model parameters, *b* and *c*, represent uncertainty and CF_intra_ represent both, two dimensional Monte Carlo simulation was performed to keep variability between consumers and uncertainty in the dose response separated [22]. For each individual in either the population or subgroups model, variability in the sensitivity to the chemical (CF_intra_) remained the same over the uncertainty iterations. In the subgroup model, the simulated values representing the uncertainty in CFs are the same for each subgroup. Simulations were performed with 10,000 and 5,000 iterations in the variability dimension and 2,000 and 1,000 iterations in the uncertainty dimension for the population risk model and subgroup risk model, respectively. The simulation algorithms for both models are given in S1 Algorithms.

## Results

**Fig 4:**
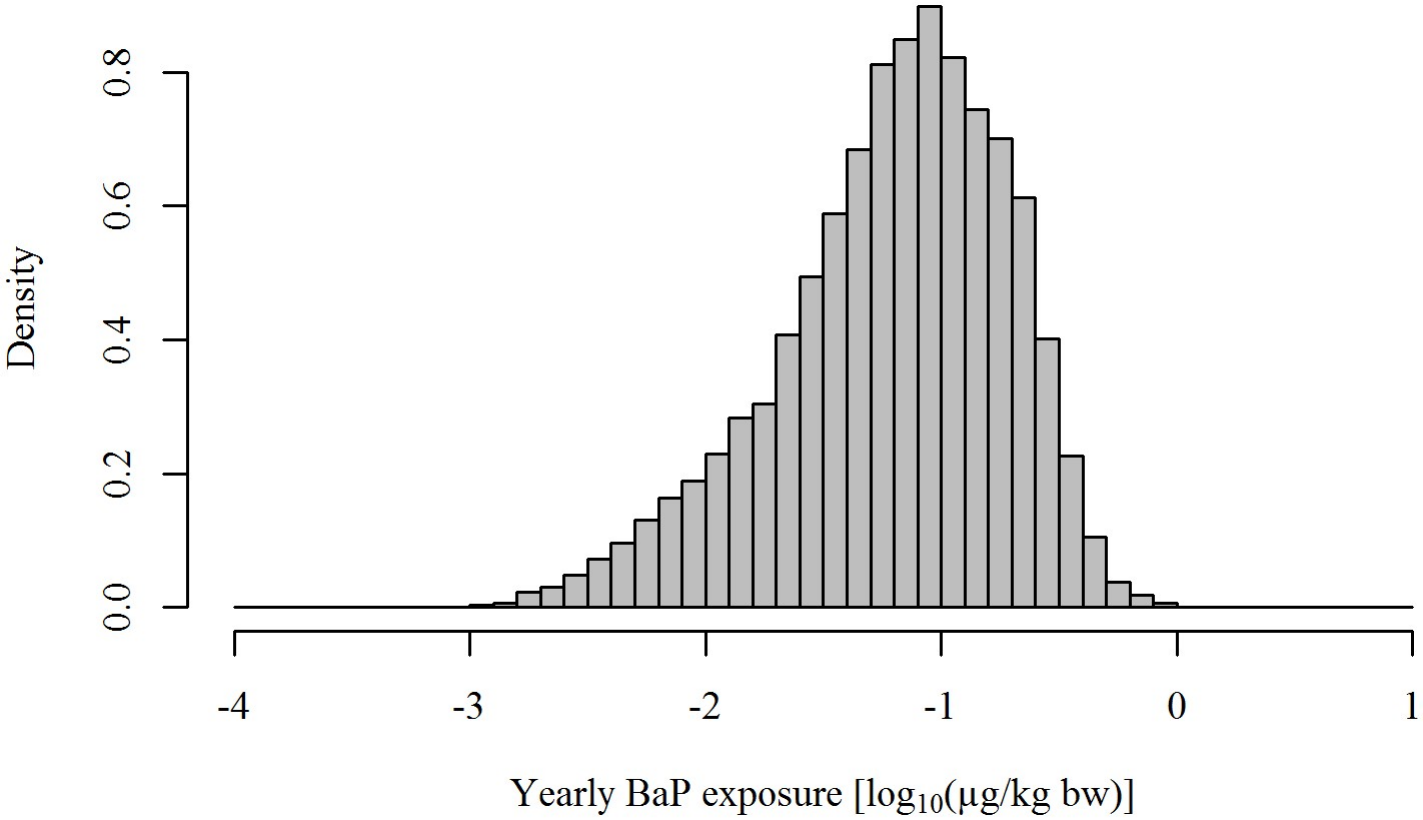
Population exposure to benzo[*a*]pyrene (in *μ*g/kgbodyweight/year) from barbecued meat on the log_10_ scale.

The simulated yearly exposure to BaP through consumption of barbecued meat in the Danish population (Eq 2) varied substantially between individuals, with a median of 0.07 *μ*g/kg bodyweight and a 95^th^ percentile of 0.29 *μ*g/kg bodyweight. Fig 4 shows the yearly exposure to BaP on a log_10_ scale for the non-zero estimated values (i.e. 96.03% of the simulated population). This reflects the variability in the population in terms of frequency and amount of consumption of barbecued meat. While most of the population is estimated to have a low yearly exposure to BaP through barbecuing, some individuals appear to be highly exposed. Individuals with exposure above -0.3 on the log_10_ scale correspond to the 97.5^th^ percentile of the Danish population.

**Fig 5:**
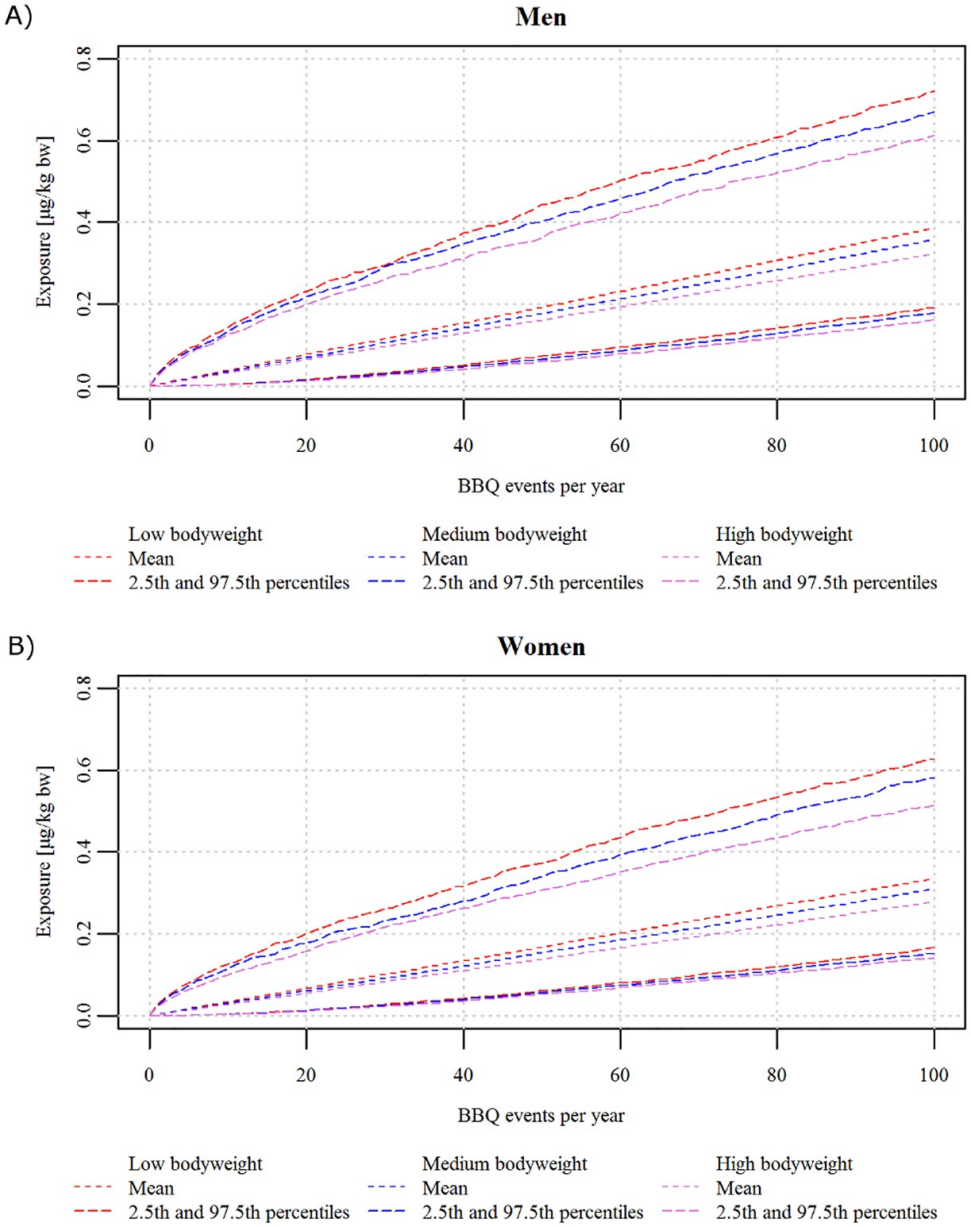
Exposure to benzo[*a*]pyrene (in *μ*g/kgbodyweight/year) as a function of events of consuming barbecued meat per year. A: Men, B: Women. Mean, 2.5^th^ and 97.5^th^ percentiles of the exposure simulated for 5,000 individuals per subgroup and per number of events from 1 to 100 per year.

With regards to the simulated exposure per barbecue event, men of low body weight had on average 9.1% and 20.2% higher exposure per event compared to men in the medium and high bodyweight classes, respectively (Fig 5A). Likewise, women in the low bodyweight class had on average 9.5% and 21.6% higher exposure per event of consuming barbecued meat compared to women belonging to the medium and high bodyweight classes, respectively (Fig 5B).

**Fig 6:**
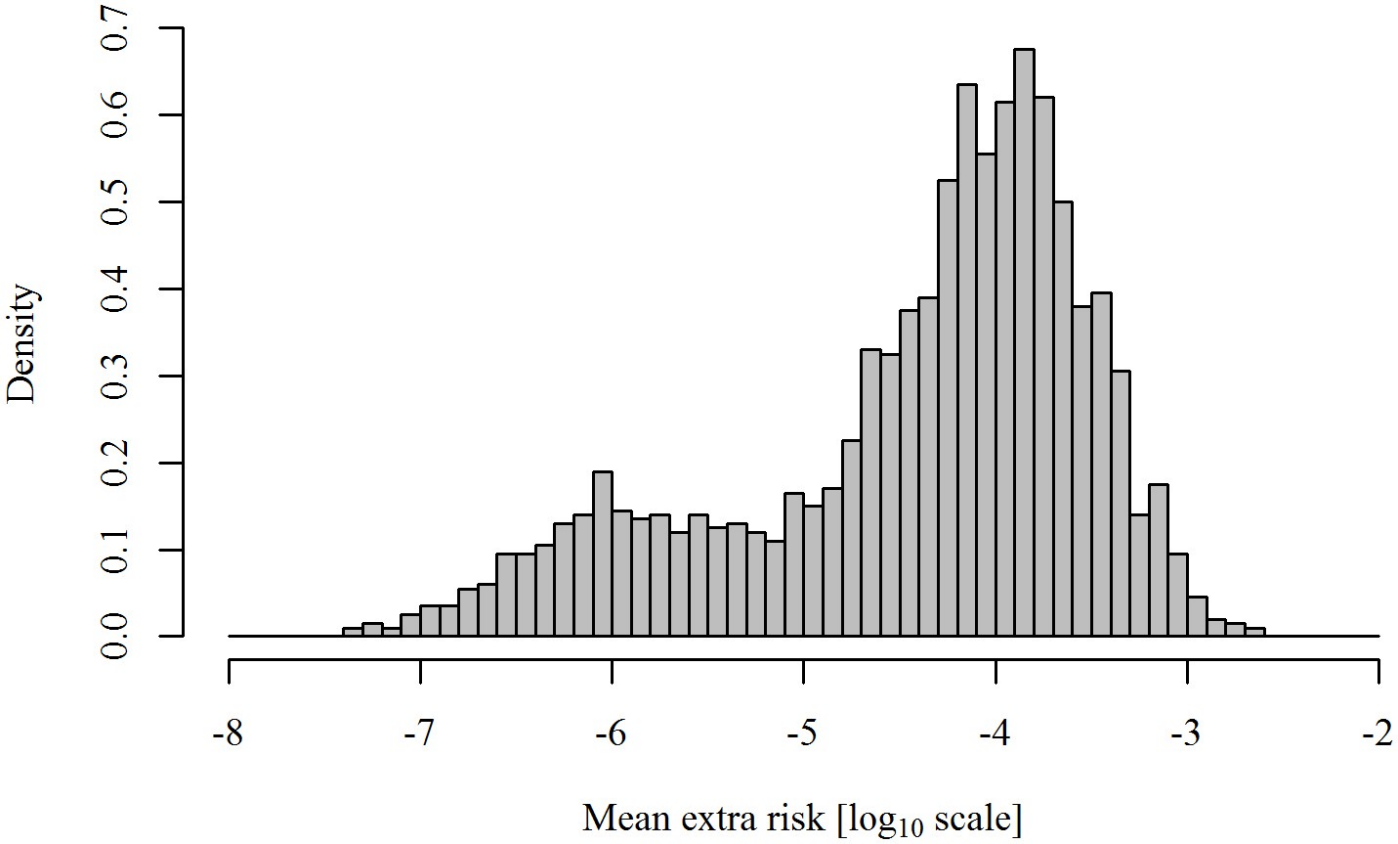
Uncertainty distribution of the mean extra lifetime risk of cancer due to exposure to benzo[*a*]pyrene from barbecued meat in the Danish population on the log_10_ scale. The distribution represents the uncertainty of the mean extra lifetime risk in the Danish population. Each value of the mean extra lifetime risk (2,000 uncertainty iterations) is the average of the 10,000 individuals (variability) for a fixed setting of the uncertainty.

The mean extra lifetime risk of cancer due to exposure to BaP from barbecued meat in the Danish population was estimated to be 6.8 × 10^−5^ (median), with a 95% uncertainty interval of 2.6 × 10^−7^ − 7.0 × 10^−4^. The uncertainty distribution of the mean extra lifetime risk on a log_10_ scale is shown in Fig 6. The range reflects the uncertainty propagated from the model extrapolation and species conversion and translates into approximately 1 to 4,074 extra cancer cases (395 for the median of the mean risk) over a lifetime in the Danish population.

**Fig 7:**
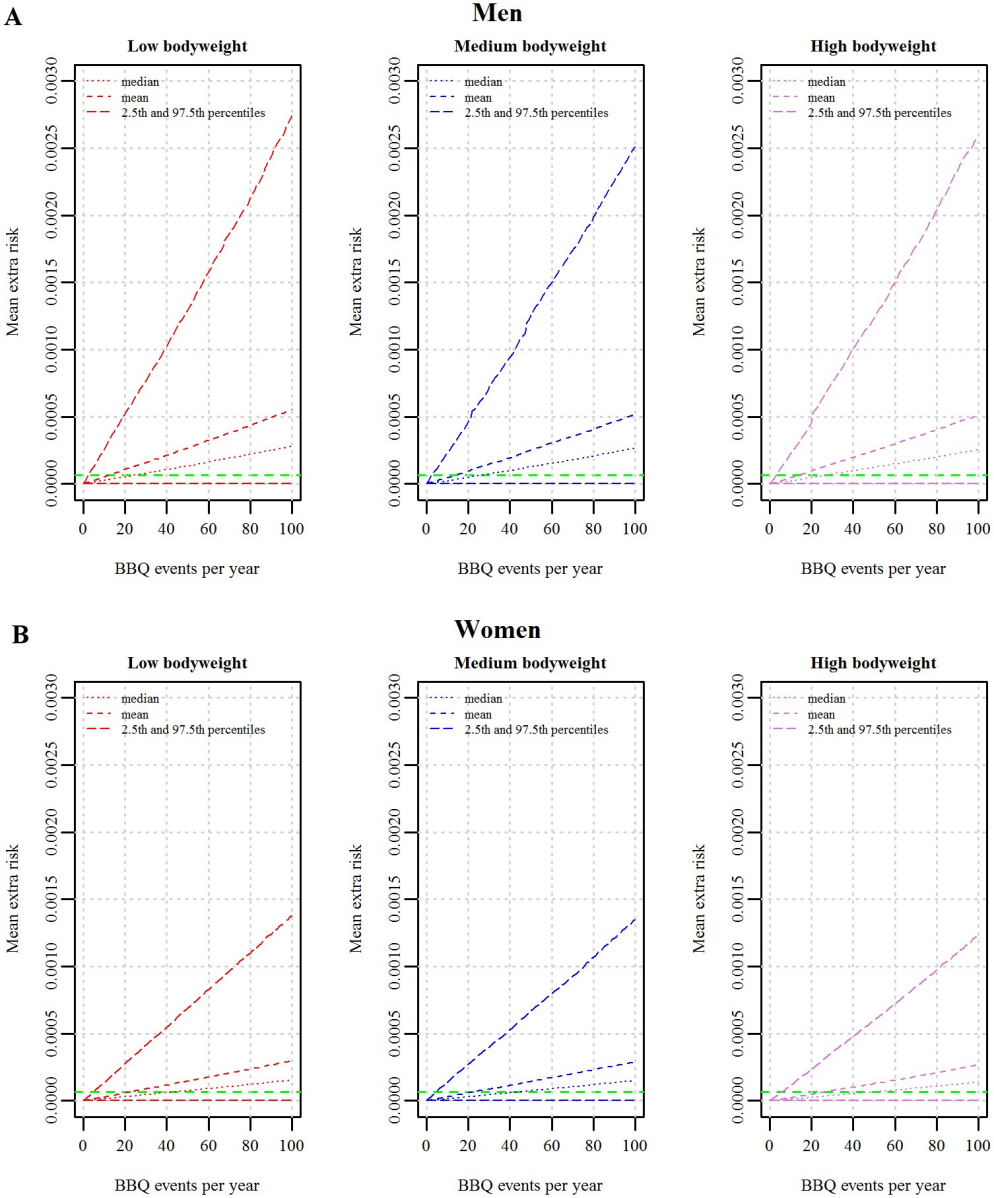
Uncertainty distribution of the mean extra lifetime risk of cancer as a function of events of consuming barbecued meat (BBQ events) for each sex and weight class. A: Men, B: Women. For each barbecue event, the uncertainty distribution of the mean risk is represented by the mean, median, 5^th^ and 95^th^ percentiles. Each value of the mean extra lifetime risk (500 uncertainty iterations) is the average of the 5,000 individuals (variability) in each subgroup for a fixed setting of the uncertainty. Green dashed lines represent the median of the mean risk from the population model.

The mean extra lifetime risk of cancer as a function of number of events of consumption of barbecued meat simulated for each sex and subgroup shows that men are at a higher risk than women of the corresponding bodyweight class, reflecting the differences in exposure (Fig 7A and 7B). The difference in risk between subgroups is attenuated by the allometric scaling. However, it is evident from our results that the uncertainty in the estimates makes the quantification of the health risk due to consumption of barbecued meat in the subgroups very imprecise, thus the variability among subgroups seems to be dwarfed by the uncertainty.

The median of the mean extra lifetime risk estimated for the total population is indicated by the dashed line in each plot of Fig 7A and 7B. The number of consumption events to exceed the mean population risk is 26, 28 and 28 per year for men of low, medium and high body weight, respectively, and 31, 34 and 40 for women of low, medium and high body weight, respectively (Table 4). In the parenthesis in Table 4, the number of events for the 97.5^th^ percentile to exceed the mean extra lifetime risk is given. It is for example seen that if men of low bodyweight consume barbecued meat three times per year they are 97.5% certain to exceed the median of the mean extra lifetime risk of the total population.

**Table 4:** Number of barbecuing events per year for the median (and 97.5^th^ percentile) of the mean extra risk for each subgroup to exceed the median of the mean population risk.

Weight class	Men	Women
Low	26 (3)	31 (3)
Medium	28 (3)	34 (4)
High	28 (4)	40 (5)

## Discussion

Our study proposes a probabilistic approach to assess cancer risk as a function of consumer attributes. In this analysis, the frequency of consuming barbecued meat was modelled by applying an event-based simulation scheme for the exposure estimation, combined with a model extrapolation approach for the hazard characterization. Our model takes into account the inter-individual variation in the exposure and sensitivity to develop cancer after exposure to BaP in the population, and quantifies the uncertainties associated with the hazard characterization.

To account for the variability in the population in terms of different factors (e.g. food consumption habits, bodyweight, etc.) makes it possible to estimate the fraction of a (sub)population that is subject to a specific risk level. By probabilistically assessing exposure in an event-based manner, our approach allows for an assessment of individuals’ current practices (in this case frequency of consuming barbecued meat) and the associated probability of an adverse health effect (in this case developing cancer).

We estimated that the extra lifetime risk of cancer due to exposure to BaP from barbecued meat in the Danish population is between 1.7 × 10^−7^ (2.5^th^ percentile) and 4.5 × 10^−4^ (97.5^th^ percentile). Thus, the uncertainty makes quantification of the cancer risk due to barbecuing in both the population and subgroup models very imprecise. The implication of this in a policy making perspective is that the assessment of the health risk from barbecuing is imperfect. To imply a precautionary principle, the (95% uncertainty) interval indicates that a risk exceeding a one in a million or one in 100,000 extra risk of cancer over a lifetime, which is typically used as an acceptable risk, cannot be excluded [6, 40]. It should further be noted that the BaP concentrations in three of the most consumed meat types (pork, sausages and minced meat (S3 and S4 Tables)) are often exceeding the legally enforced maximum limit of 5 *μ*g/kg (Fig 3).

By combining the population and subgroup models, we found that e.g. men and women of low bodyweight consuming barbecued meat 26 and 31 times per year, respectively, are above the median of the mean population risk (Table 4). However, information on the consumption frequency to exceed any other risk level of interest (e.g. the 95^th^ percentile of the mean population risk, or a fixed risk level such as one in a million) may be derived from the model. Again, the variability between subgroups seems to be dwarfed by the large uncertainty.

The uncertainty interval only reflects the uncertainty propagated from the two-stage dose-response model and the inter- and intra-species conversion. Applying the model extrapolation approach (contrary a linear extrapolation) allowed for propagating the uncertainty in the cancer risk estimation; propagating the model uncertainty and the uncertainty in the exposure would improve our study, but would also naturally add to the range of uncertainty.

Our results support the opinion of the European Food Safety Agency that a (precise, i.e. with a small uncertainty interval) cancer risk estimate cannot be derived when low risks are concerned, why cancer risks from chemical dietary exposures in the European Union are now assessed by a Margin of Exposure (MOE) approach (i.e. a ratio of the BMDL to the observed exposure below 10,000 constitutes a concern to human health) [40–42].

### Comparison with other studies

Other exposure and risk assessments of BaP exposure from food including barbecued meat have been performed e.g. [43, 44]. In the two most relevant studies conducted in Scandinavia, population exposure to BaP was assessed deterministically for Sweden [32] and Norway [45], both assuming a 70 kg bodyweight. Exposure to BaP from barbecued meat to be 0.0062 *μ*g/kg bodyweight per year and 2.665 *μ*g/kg bodyweight per year. These values are approximately 11 times lower and 38 times higher, respectively, than our exposure estimate (0.07 *μ*g/kg bodyweight per year). The discrepancies in exposure among these three studies can be explained by the different model assumptions in the number of events per year, the amounts of meat consumed per event and the BaP concentrations in the consumed meat. The Norwegian study applied a conservative approach where BaP concentrations in meat types where intended to be the highest identified in the literature [45]; which most likely results in the 38 times higher exposure estimate.

No quantitative estimates of the risk were reported in [32] and [45], however MOEs of 5,000,000 and 13,611, respectively, were calculated using the above reported exposures (expressed per day) and a BMDL for a 10% incidence of 100 *μ*g/kg bodyweight/day as estimated by JECFA [6] based on the same animal study that we used. If assuming a linear extrapolation from JECFA’s BMDL, the BaP exposures in [32] and [45] result in risk estimates of less than 10^−7^ and 7.3 × 10^−6^, respectively. It should be noted that the latter is one ninth lower than our estimated median of the mean extra lifetime risk (6.8 × 10^−5^) despite the exposure in [45] being 38 times higher and the assumption of the linear extrapolation, which is usually considered a conservative approach. This can partly be explained by the approach and assumptions applied in JECFAs modelling of the BMDL; i.e. based on the same animal study, EFSA arrived at a BMDL of 70 *μ*g/kg bodyweight/day [7], from which a risk of 1.04 × 10^−5^ can be derived by linear extrapolation. The risk estimate is still within our 95% uncertainty interval, but indicates that an assumption of linearity below the observable dose range is not necessarily conservative, highlighting the importance of quantifying the uncertainty in the dose response relationship, as also suggested in [18].

Another recent study also presented probabilistic estimates of concentration of PAH’s including BaP in meat and bread, predicted by use of a meta-analysis approach in which key factors (e.g. cooking method, time, temperature, meat cuts etc.) influencing the formation of the compounds were identified in the literature, analysed and taken into account [46]. While it is very relevant to take those factors into account in exposure modelling of process contaminants, the advantage of our concentration datasets is that they are obtained from a wide range of barbecue settings, both private, commercial and experimental, and thus the fitted distributions should represent the possible range of concentrations of BaP in barbecued meat and reflect the influence of various factors. However, if such dataset is not available, the approach proposed in [46] is recommended. The concentration levels predicted in [46] were combined with consumption data to estimate exposure in the US population [47]; exposure to BaP from barbecued beef was reported to 0.15 *μ*g/kg bw/year, assuming a 70 kg bodyweight, which is considerably higher than our estimate.

It is relevant to discuss our findings in relation to epidemiological evidence on the association between BaP and/or barbecued meat consumption and the risk of cancer. However, this evidence is sparse. To our knowledge, a single case control study identified a positive statistical significant association between BaP in processed red meat and rectal cancer, however no quantification of the risk by the incremental increase in exposure was reported [48]. The same study did not find an association between colorectal cancer and exposure to grilled/barbecued meat. It has been speculated whether the statistical power in observational studies is large enough to be able to detect cancer risks in the same magnitude as our results, especially when exposures occur via mulitple routes [49]. The multi-causal nature of cancer may hamper the ability to detect a statistical significant association between a single compound or cooking method, also taking into account residual confounding and the risk of misclassification bias [50].

### Assumptions and limitations

Due to lack of data, several assumptions were made in this study. These may affect the representativeness of the input distributions, but were not translated into a quantitative estimate of uncertainty. All assumptions are listed in Table 5, as is the potential impact these assumption may have on the estimations.

**Table 5:** List of assumptions and potential impact on the estimates.

Data source	Assumption	Potential impact on final estimates	Reference
Concentration data	[BaP] is independent on the weight of meat consumed. However, high weight of meat eaten = long barbecue time = high [BaP]	Likely underestimation of [BaP] in large portion sizes	[27, 29, 31, 46]
	[BaP] is independent on the fat type and content. However, high fat content = high [BaP]	Likely underestimation of [BaP] in fatty meat	
	Type of and distance to heating source affect [BaP], but is unknown.	Unknown if leading to under- or overestimation of [BaP]	
Consumption data	People eat the same amount of meat when barbecuing compared to a non-barbecue eating occasion.	Likely underestimation of the meat consumption	[25]
Dose-response data	The total potency of the cumulative effect of all PAHs in the coal-tar mixtures is the same as for BaP	Likely overestimation of the potency of BaP	[37]
	Two-stage model describe the dose response relationship	Likely overestimation of lifetime cancer risk	
	No components of the meat (e.g. linoleic acid) or in the accompanying diet (e.g. antioxidants) inhibits the effects of BaP	Likely overestimation of the lifetime cancer risk	[51]

Besides the listed assumptions, our study suffered from various limitations. An important limitation in the population model is the data used to inform on the frequency of consuming barbecued meat in the Danish population. These data are only based on qualitative information from 76 families living in a suburbian area (in houses rather than highrise buildings) and we do not know how representative the sample is of the total Danish population. Besides, we assumed that only vegetarians (estimated 4% of the Danish population) do not consume barbecued meat. However, the proportion of the population not consuming barbecued meat may very well be influenced by other factors such as access to a barbecue etc. For comparison, a Norwegian survey on barbecuing behaviour including 1,003 participants reported 26% barbecuing more than 17 times/year, 34% barbecuing 6-17 times/year and 27% barbecuing less than 6 times per year [45]. In comparison, our data seem to overestimate the barbecuing frequency and thus also the population risk. To our knowledge, no other data on the frequency of consuming barbecued meat in the Danish population are available. Diets change over time and will impact the lifetime risk of individuals. The consumption data derived from DANSDA do not reflect this variation over time of the individual, which is a limitation of the dataset. However, we simulate the variation in the annual exposure of the (sub)populations, which then represents a snapshot of the (sub)population’s exposure. In this way the change in diet over time is taken into account and is reflected in the extra life time risk of the population.

In Eq (1), which determines the weight of the simulated individual based on sex and age, there is a tendency of the residuals not being completely symmetrical, which means that the variability in the generated population is not fully accounted for. Also other factors besides sex and age could be considered when estimating an individuals weight, e.g. height.

Furthermore, our models suffered from the challenge of combining data from a vast range of sources. This inflicts inconsistency e.g. in the categorization of meat types consumed and meat types sampled for BaP content. Also, this combination of datasets adds to the overall uncertainty of the final estimates.

We only considered BaP in this study. However, a variety of carcinogenic PAHs are formed during barbecuing, together with other carcinogenic compounds such as heterocyclic aromatic amines, and thus the risk of cancer due to consumption of barbecued meat is likely higher [52]. EFSA and others have concluded that BaP is not a good surrogate for other PAH’s [7, 47], so probabilistic modelling of each of the other PAHs would be necessary to include them in our study. We also did not consider exposure to BaP (or other PAHs) from other sources. The main sources of BaP and other PAHs from foods in Denmark are cereals, vegetables and milk; the mean exposure from all food sources is 1.41 *μ*g/kg bodyweight per year, assuming a 70 kg bodyweight [28]. This suggests that the contribution of barbecued meat to the total dietary exposure is approximately 5%. Smoking and air pollution are other major sources of BaP exposure [53]. Therefore, other dietary sources, smoking and the environment likely contribute greatly to the cancer risk. Since our dose response relationship is not linear, it would be relevant to assess the “acceptable” frequency of consuming barbecued meat taking these background exposures into consideration. Likewise, a risk assessment of the aggregated exposure to PAH and other carcinogenic compounds in barbecued meat would give a more comprehensive estimate of the cancer risk; an assessment of the risk from other food sources via our proposed event-based simulation approach would improve our study and provide valuable information on the relative contribution to the risk of cancer in the various subpopulations from different foodgroups.

### Future application of the model

Our model defined subgroups by predicting meat consumption based on information on sex, age and bodyweight obtained from the Danish national food consumption survey. In future applications, the approach can be adapted to further define subgroups on the basis of other characteristics (e.g. socio-economics status, genetics, etc.). Because this approach is able to quantify and characterize the variability in a population, it allows for the formulation of mitigation strategies that are targeted to subgroups of higher risk. It also allows for more effective risk communication, which can be customized to these specific groups by accounting for their current behaviour [12, 54].

## Conclusion

This study showed that the risk of cancer due to exposure to BaP in barbecued meat may not be negligible for highly exposed individuals. The proposed model can be applied to other assessments and allows for deriving the change in risk following a specific change in behaviour. We argue that in the future it can serve as a valuable tool for risk managers as it enables them to advise a change in behaviour according to a specific risk level that may be found acceptable in a given population.

## Supporting information

S1 Fig Age and sex demographics of the Danish population. (PDF)

S1 Table Parameters for the gamma distributions of meat consumption. (PDF)

S2 Table Frequency of consuming one or two types of meat per eating occasion. (PDF)

S3 Table Frequency of type of meat consumed, if one meat per meal is consumed. (PDF)

S4 Table Frequency of type of meat and fraction of total meat consumed, if two meat types are consumed. (PDF)

S5 Table Parameters for the censored log-normal distributions of concentration of Bap in barbecued meat. (PDF)

S6 Table Dose response data of BaP in coal tar mixtures and tumor bearing mice from [36] as reported in [37] and [7]. (PDF)

S1 Algorithms Simulation models. (PDF)
