# Supplementary material for: Probabilistic approach for assessing cancer risk due to benzo[a]pyrene in barbecued meat: Informing advice for population groups
Source: PLoS One. 2018 Nov 8;13(11):e0207032. doi: 10.1371/journal.pone.0207032 (PMC6224084; doi:10.1371/journal.pone.0207032)
Supplement: S1 Fig — (PDF) [file pone.0207032.s001.pdf]

S1 Fig

Age and sex demographics of the Danish population

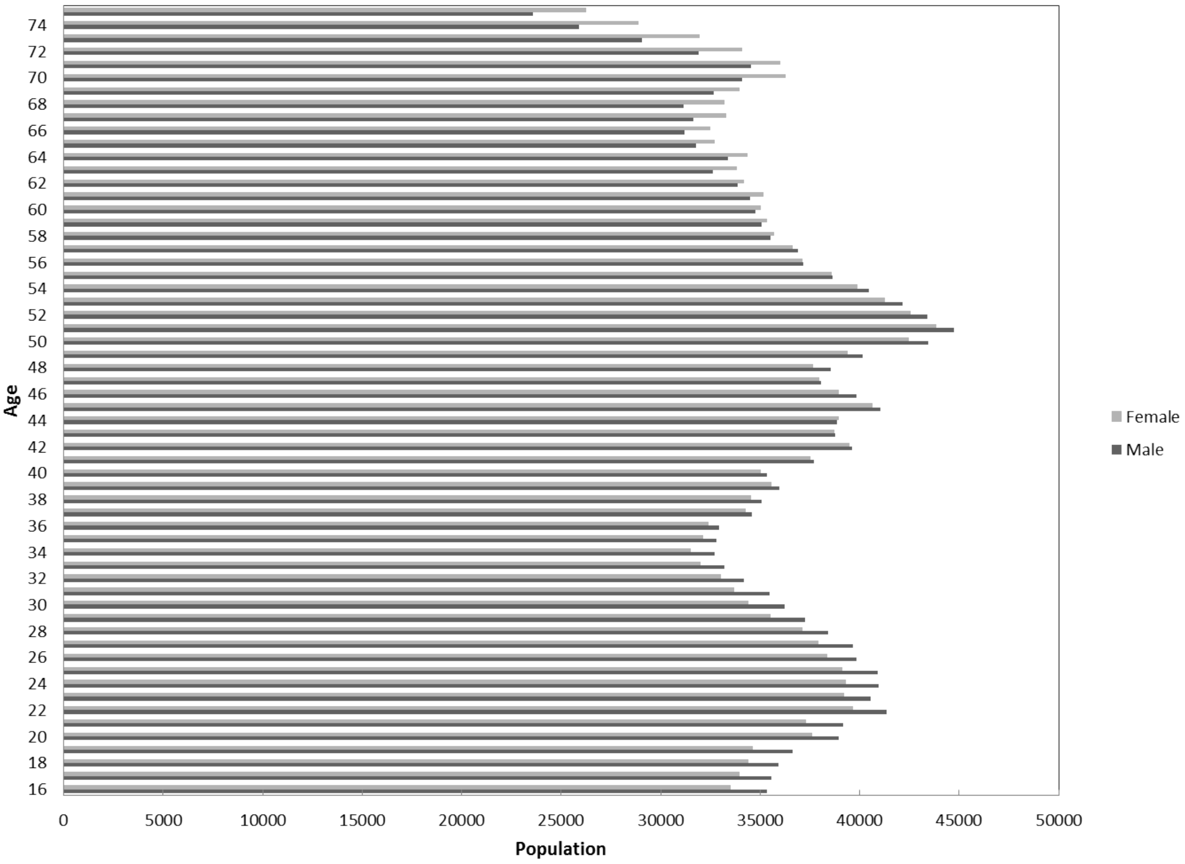

Figure 1: Population size of males and females between age 16-75 in the Danish population per 1st day in the 4th quater of 2017 (Statistics Denmark, 2018, www.statistiskbanken.dk/FOLK1A)
