# Supplementary material for: Probabilistic approach for assessing cancer risk due to benzo[a]pyrene in barbecued meat: Informing advice for population groups
Source: PLoS One. 2018 Nov 8;13(11):e0207032. doi: 10.1371/journal.pone.0207032 (PMC6224084; doi:10.1371/journal.pone.0207032)
Supplement: S1 Table — (PDF) [file pone.0207032.s002.pdf]

S1 Table

Parameters for the gamma distribution of meat consumption
Gamma distribution, Gamma(α, β) from which the consumption of meat in g/meal is derived.

Weight class	Males	Females
Low	(2.551, 0.016)	(2.483, 0.022)
Medium	(2.549, 0.015)	(2.649, 0.022)
High	(2.513, 0.014)	(2.829, 0.022)
