# Supplementary material for: Probabilistic approach for assessing cancer risk due to benzo[a]pyrene in barbecued meat: Informing advice for population groups
Source: PLoS One. 2018 Nov 8;13(11):e0207032. doi: 10.1371/journal.pone.0207032 (PMC6224084; doi:10.1371/journal.pone.0207032)
Supplement: S2 Table — (PDF) [file pone.0207032.s003.pdf]

S2 Table

Frequency of consuming one or two types of meat per eating occasion.

**Number of meat types**	**One meat type**	**Two meat types**
**Frequency**	0.260	0.740
