# Supplementary material for: Probabilistic approach for assessing cancer risk due to benzo[a]pyrene in barbecued meat: Informing advice for population groups
Source: PLoS One. 2018 Nov 8;13(11):e0207032. doi: 10.1371/journal.pone.0207032 (PMC6224084; doi:10.1371/journal.pone.0207032)
Supplement: S3 Table — (PDF) [file pone.0207032.s004.pdf]

S3 Table

Probability of type of meat consumed, if one meat per meal is consumed

Meat type	Beef	Minced beef	Pork	Sausages	Lamb	Poultry	Fish	Shellfish
**Frequency**	0.1285	0.1186	0.1839	0.3448	0.0230	0.1609	0.0266	0.0137
