# Supplementary material for: Probabilistic approach for assessing cancer risk due to benzo[a]pyrene in barbecued meat: Informing advice for population groups
Source: PLoS One. 2018 Nov 8;13(11):e0207032. doi: 10.1371/journal.pone.0207032 (PMC6224084; doi:10.1371/journal.pone.0207032)
Supplement: S4 Table — (PDF) [file pone.0207032.s005.pdf]

S4 Table

Probability of types of meat if two meats are consumed

Probability of combinations of two meat types consumed together at a meal (fraction of each meat type out of the total amount consumed is given in parenthesis).

	Beef	Minced beef	Pork	Sausages	Lamb	Poultry	Fish	Shellfish
**Beef**		0.0348<br>(0.5192, 0.4808)	0.0561<br>(0.5906, 0.4094)	0.0484<br>(0.5387, 0.4613)	0.0064<br>(0.6154, 0.4505)	0.0484<br>(0.5485, 0.4505)	0.0074<br>(0.6067, 0.3933)	0.0038<br>(0.6695, 0.3305)
**Minced beef**			0.0515<br>(0.7282, 0.2718)	0.1088<br>(0.4759, 0.5948)	0.0059<br>(0.5217, 0.4783)	0.0444<br>(0.5750, 0.4250)	0.0068<br>(0.5852, 0.4148)	0.0035<br>(0.8042, 0.1958)
**Pork**				0.1745<br>(0.4989, 0.5011)	0.0095<br>(0.5559, 0.4441)	0.0715<br>(0.4009, 0.5991)	0.0110<br>(0.4565, 0.5435)	0.0056<br>(0.6780, 0.3292)
**Sausages**					0.0202<br>(0.6840, 0.3160)	0.1508<br>(0.5044, 0.4956)	0.0234<br>(0.5116, 0.4884)	0.0120<br>(0.6364, 0.3636)
**Lamb**						0.0082<br>(0.5176, 0.4824)	0.0013<br>(0.5242, 0.4758)	0.0006<br>(0.6772, 0.3228)
**Poultry**							0.0095<br>(0.4476, 0.5524)	0.0049<br>(0.5844, 0.4156)
**Fish**								0.0007<br>(0.6647, 0.3353)
**Shellfish**								
