# Supplementary material for: Probabilistic approach for assessing cancer risk due to benzo[a]pyrene in barbecued meat: Informing advice for population groups
Source: PLoS One. 2018 Nov 8;13(11):e0207032. doi: 10.1371/journal.pone.0207032 (PMC6224084; doi:10.1371/journal.pone.0207032)
Supplement: S5 Table — (PDF) [file pone.0207032.s006.pdf]

S5 Table

Parameters for the log-normal distributions of the concentration of Bap in barbecued meat
Log-normal distributions with mean, μ, and standard deviation, σ, from which the concentration of Bap in μg/kg in each meat type after barbecuing is derived.
$^{a)}$ For the minced beef, we applied a 2-component log-normal model on the data, where the mixing proportions were estimated to be (0.300, 0.700). The optimization technique used for minced beef is the maximum a posterior posterior (MaP) estimation (Fraley & Raftery, 2007)[1].
$^{b)}$ Means for the mixture model.
$^{c)}$ Standard deviations for the mixture model.

**Meat**	**Samples**	$\leq$ **LOD**	$\geq$ **LOD**	$\boldsymbol{\mu}$	$\boldsymbol{\sigma}$
Beaf	87	44	43	-2.326	1.626
Minced beaf$^{a)}$	78	28	50	(2.629, -2.123)$^{b)}$	(0.430, 2.307)$^{c)}$
Pork	70	39	31	-2.932	2.419
Sausages	58	8	50	-1.141	1.991
Lamb	15	2	13	-0.712	1.484
Poultry	53	23	30	-2.134	1.368
Fish	42	13	29	-1.518	1.599
Shellfish	4	0	4	-1.783	0.575

[1] Fraley, C., Raftery, A.E. Bayesian regularization for normal mixture estimation and model-based clustering. Journal of Classification. 2007; 24(2), pp 155-181. doi: 10.1007/s00357-007-0004-z
