# Supplementary material for: Probabilistic approach for assessing cancer risk due to benzo[a]pyrene in barbecued meat: Informing advice for population groups
Source: PLoS One. 2018 Nov 8;13(11):e0207032. doi: 10.1371/journal.pone.0207032 (PMC6224084; doi:10.1371/journal.pone.0207032)
Supplement: S6 Table — (PDF) [file pone.0207032.s007.pdf]

S6 Table

Data used for dose response modelling

Number of tumor bearing mice per dose group of BaP in coal tar mixtures. Response is number of mice with tumors after 2 years (an animal lifetime) of exposure to BaP. Dosage is expressed in μg per kg bodyweight per year extrapolated from the data reported by Schneider et al. (2002)[1], taking into account the ratio between animal and human lifetime.

Coal tar mixture	BaP dose (μg/kg bw/year)	Number of tumor bearing animals	Sample size
1	0	5	48
1	9855	12	48
1	28835	14	48
1	97090	12	48
1	287985	40	48
2	44165	17	48
2	160600	23	48
2	408800	44	48

[1] Schneider, K., Roller, M., Kalberlah, F., Schuhmacher-Wolz, U. Cancer risk assessment for oral exposure to PAH mixtures. Journal of Applied Toxicology. 2002; 22(1):73-83. doi:10.1002/jat.828
