# Supplementary material for: Probabilistic approach for assessing cancer risk due to benzo[a]pyrene in barbecued meat: Informing advice for population groups
Source: PLoS One. 2018 Nov 8;13(11):e0207032. doi: 10.1371/journal.pone.0207032 (PMC6224084; doi:10.1371/journal.pone.0207032)
Supplement: S1 Algorithms — (PDF) [file pone.0207032.s008.pdf]

S1 Algorithms

In this appendix, the following notation is used:

$N_{\text{population}}$: number of individuals in population model (10,000 in this study);
$N_{\text{subgroups}}$: number of individuals in each population subgroup (5,000 in this study);
N_{unc}: number of uncertainty iterations (1,000 and 500 for the population model and the subgroups model, respectively);
ML: men of low body weight;
MM: men of medium body weight;
MH: men of high body weight;
WL: women of low body weight;
WM: women of medium body weight;
WH: women of high body weight.

Algorithm 1 Population exposure simulation

```
for each individual i ∈ {1, . . . , N_population} do
    generate a r.v. s_i for the sex of individual i
    generate a r.v. a_i for the age of individual i
    estimate the weight w_i of individual i
    assign individual i to a weight class c
    generate a r.v. B_i for the barbecuing scenario of individual i
    for each event b ∈ {1, . . . , B_i} do
        generate a r.v. t_ib for the total meat consumption, given the class c of the individual i
        generate a r.v. K_ib for the total number of consumed meat types
        for each meat type k ∈ {1, . . . , K_ib} do
            generate a r.v. m_ibk for the consumed meat type k
            generate a r.v. v_ibk for the fractions of consumed meat type m_ibk
            calculate the ammount x_ibk of consumed meat type m_ibk, i.e. x_ibk = t_ib · v_ibk
            generate a r.v. c_ibk for the BaP concentration in the consumed meat type m_ibk
        end for
    end for
    calculate the BaP exposure y_i, i.e. y_i = (1/w_i) Σ_{b=1}^{B_i} Σ_{k=1}^{K_ib} x_ikb · c_ibk
end for
```

Algorithm 2 Population cancer risk simulation

for each individual $i \in \{1, \ldots, \mathrm{N}_{\mathrm{population}}\}$ **do**
  generate a uniform r.v. U_i
end for
for each event $n \in \{1, \ldots, \mathrm{N}_{\mathrm{unc}}\}$ **do**
  generate a r.v. $\mathrm{AP}^{(n)}$
  calculate $\mathrm{CF}^{(n)}_{\mathrm{inter,allometric}}$
  generate a r.v. $\mathrm{CF}^{(n)}_{\mathrm{inter,TKTD}}$
  generate a r.v. $\mathrm{GSD}^{(n)}_{\mathrm{CF_{intra}}}$
  generate a bivariate r.v. $(b^{(n)}, c^{(n)})$
  for each individual $i \in \{1, \ldots, \mathrm{N}_{\mathrm{population}}\}$ **do**
    calculate $\mathrm{CF}^{(n)}_{\mathrm{intra},i}$ using the inverse transformation method, i.e.

$$\mathrm{CF}^{(n)}_{\mathrm{intra},i} = \exp\left(\ln\left(\mathrm{GM}_{\mathrm{CF_{intra}}}\right) + \ln\left(\mathrm{GSD}_{\mathrm{CF_{intra}}}\right) \cdot \sqrt{21/\mathrm{GSD}^{(n)}_{\mathrm{CF_{intra}}}} \cdot \Phi^{-1}\left(U_i\right)\right)$$

    calculate the animal exposure $\exp^{(n)}_{\mathrm{animal},i}$, i.e.

$$\exp^{(n)}_{\mathrm{animal},i} = y_i \cdot \mathrm{CF}^{(n)}_{\mathrm{intra},i} \cdot \mathrm{CF}^{(n)}_{\mathrm{inter,allometric}} \cdot \mathrm{CF}^{(n)}_{\mathrm{inter,TKTD}}$$

    calculate the extra lifetime risk $\mathrm{ER}^{(n)}_{\mathrm{BaP},i}$, i.e.

$$\mathrm{ER}^{(n)}_{\mathrm{BaP},i} = 1 - \exp\left(\left(\frac{\exp^{(n)}_{\mathrm{animal},i}}{b}\right) - c\left(\frac{\exp^{(n)}_{\mathrm{animal},i}}{b}\right)^2\right)$$

  end for
end for

Algorithm 3 Subgroup exposure simulation

for each class $c \in \{\mathrm{ML,MM,MH,WL,WM,WH}\}$ **do**
  for each individual $i \in \{1, \ldots, \mathrm{N_{subgroups}}\}$ **do**
    for each event $b \in \{1, \ldots, \mathrm{N_{bbq}}\}$ **do**
      generate a r.v. $t_{ib}(c)$ for the total meat consumption
      generate a r.v. $K_{ib}(c)$ for the total number of consumed meat types
      for each meat type $k \in \{1, \ldots, K_{ib}(c)\}$ **do**
        generate a r.v. $m_{ibk}(c)$ for the consumed meat type
        generate a r.v. $v_{ibk}(c)$ for the fractions of consumed meat type $m_{ibk}(c)$
        calculate the ammount $x_{ibk}(c)$ of consumed meat type $m_{ibk}(c)$, i.e. $x_{ibk}(c) = t_{ib}(c) \cdot v_{ibk}(c)$
        generate a r.v. $c_{ibk}(c)$ for the BaP concentration in the consumed meat type $m_{ibk}(c)$
      end for
      calculate the BaP intake $y_{ib}(c)$, i.e. $y_{ib}(c) = \sum_{k=1}^{K_{ib}(c)} x_{ibk}(c) \cdot c_{ibk}(c)$ at event b
    end for
    for each event $B_i(c) \in \{1, \ldots, \mathrm{N_{bbq}}\}$ **do**
      calculate the BaP exposure $y_i(c, B)$, i.e. $y_i(c, B) = \frac{1}{w(c)} \sum_{b=1}^{B_i(c)} y_{ib}(c)$, where $w(c)$ is the median bodyweight of class c
    end for
  end for
end for

Algorithm 4 Subgroup cancer risk simulation

for each class $c \in \{\text{ML,MM,MH,WL,WM,WH}\}$ **do**
  for each individual $i \in \{1, \ldots, \text{N}_{\text{subgroups}}\}$ **do**
    generate a uniform r.v. $U_i(c)$
  end for
end for
for each event $n \in \{1, \ldots, \text{N}_{\text{unc}}\}$ **do**
  generate a r.v. $\text{AP}^{(n)}$
  generate a r.v. $\text{CF}^{(n)}_{\text{inter,TKTD}}$
  generate a r.v. $\text{GSD}^{(n)}_{\text{CF}_{\text{intra}}}$
  generate a bivariate r.v. (b^n, c^n)
  for each class $c \in \{\text{ML,MM,MH,WL,WM,WH}\}$ **do**
    calculate $\text{CF}^{(n)}_{\text{inter,allometric}}(c)$
    for each individual $i \in \{1, \ldots, \text{N}_{\text{subgroups}}\}$ **do**
      calculate $\text{CF}^{(n)}_{\text{intra},i}(c)$ using the inverse transformation method, i.e.

$$\text{CF}^{(n)}_{\text{intra},i}(c) = \exp\left(\ln\left(\text{GM}_{\text{CF}_{\text{intra}}}\right) + \ln\left(\text{GSD}_{\text{CF}_{\text{intra}}}\right) \cdot \sqrt{21/\text{GSD}^{(n)}_{\text{CF}_{\text{intra}}}} \cdot \Phi^{-1}\left(U_i(c)\right)\right)$$

      for each event $B \in \{1, \ldots, \text{N}_{\text{bbq}}\}$ **do**
        calculate the animal exposure $\exp^{(n)}_{\text{animal},i}(c, B)$, i.e.

$$\exp^{(n)}_{\text{animal},i}(c, B) = y_i(c, B) \cdot \text{CF}^{(n)}_{\text{intra},i}(c) \cdot \text{CF}^{(n)}_{\text{inter,allometric}}(c) \cdot \text{CF}^{(n)}_{\text{inter,TKTD}}$$

        calculate the extra lifetime risk $\text{ER}^{(n)}_{\text{BaP},i}(c, B)$, i.e.

$$\text{ER}^{(n)}_{\text{BaP},i}(c, B) = 1 - \exp\left(\left(\frac{\exp^{(n)}_{\text{animal},i}(c, B)}{b}\right) - c\left(\frac{\exp^{(n)}_{\text{animal},i}(c, B)}{b}\right)^2\right)$$

      end for
    end for
  end for
end for
